# Microbially-Enhanced Vanadium Mining and Bioremediation Under Micro- and Mars Gravity on the International Space Station

**DOI:** 10.3389/fmicb.2021.641387

**Published:** 2021-04-01

**Authors:** Charles S. Cockell, Rosa Santomartino, Kai Finster, Annemiek C. Waajen, Natasha Nicholson, Claire-Marie Loudon, Lorna J. Eades, Ralf Moeller, Petra Rettberg, Felix M. Fuchs, Rob Van Houdt, Natalie Leys, Ilse Coninx, Jason Hatton, Luca Parmitano, Jutta Krause, Andrea Koehler, Nicol Caplin, Lobke Zuijderduijn, Alessandro Mariani, Stefano Pellari, Fabrizio Carubia, Giacomo Luciani, Michele Balsamo, Valfredo Zolesi, Jon Ochoa, Pia Sen, James A. J. Watt, Jeannine Doswald-Winkler, Magdalena Herová, Bernd Rattenbacher, Jennifer Wadsworth, R. Craig Everroad, René Demets

**Affiliations:** ^1^UK Centre for Astrobiology, School of Physics and Astronomy, University of Edinburgh, Edinburgh, United Kingdom; ^2^Department of Biology – Microbiology, Aarhus University, Aarhus, Denmark; ^3^School of Chemistry, University of Edinburgh, Edinburgh, United Kingdom; ^4^Radiation Biology Department, German Aerospace Center (DLR), Institute of Aerospace Medicine, Köln, Germany; ^5^Institute of Electrical Engineering and Plasma Technology, Faculty of Electrical Engineering and Information Sciences, Ruhr University Bochum, Bochum, Germany; ^6^Microbiology Unit, Belgian Nuclear Research Centre, SCK CEN, Mol, Belgium; ^7^ESTEC, Noordwijk, Netherlands; ^8^Kayser Italia S.r.l., Livorno, Italy; ^9^Space Application Services NV/SA, Noordwijk, Netherlands; ^10^Earth and Environmental Sciences Department, Rutgers University, Newark, NJ, United States; ^11^School of Geosciences, University of Edinburgh, Edinburgh, United Kingdom; ^12^BIOTESC, Hochschule Luzern Technik & Architektur, Lucerne School of Engineering and Architecture, Hergiswil, Switzerland; ^13^Exobiology Branch, NASA Ames Research Center, Moffett Field, CA, United States

**Keywords:** biomining, bioremediation, ISRU, vanadium, space, Mars, bioproduction, space microbiology

## Abstract

As humans explore and settle in space, they will need to mine elements to support industries such as manufacturing and construction. In preparation for the establishment of permanent human settlements across the Solar System, we conducted the ESA BioRock experiment on board the International Space Station to investigate whether biological mining could be accomplished under extraterrestrial gravity conditions. We tested the hypothesis that the gravity (*g*) level influenced the efficacy with which biomining could be achieved from basalt, an abundant material on the Moon and Mars, by quantifying bioleaching by three different microorganisms under microgravity, simulated Mars and Earth gravitational conditions. One element of interest in mining is vanadium (V), which is added to steel to fabricate high strength, corrosion-resistant structural materials for buildings, transportation, tools and other applications. The results showed that *Sphingomonas desiccabilis* and *Bacillus subtilis* enhanced the leaching of vanadium under the three gravity conditions compared to sterile controls by 184.92 to 283.22%, respectively. Gravity did not have a significant effect on mean leaching, thus showing the potential for biomining on Solar System objects with diverse gravitational conditions. Our results demonstrate the potential to use microorganisms to conduct elemental mining and other bioindustrial processes in space locations with non-1 × *g* gravity. These same principles apply to extraterrestrial bioremediation and elemental recycling beyond Earth.

## Introduction

To move permanently into space, we must be able to implement industrial processes that can support settlement, such as mining natural resources. Microorganisms are used in a variety of industrial processes on Earth (Taunton et al., 2000; Schulz et al., 2013; Druschel and Kappler, 2015; Kalev and Toor, 2018). Prominent among them is biomining (Rawlings and Silver, 1995; Johnson, 2014) in which microorganisms, and also plants, are used to enhance the release of elements such as gold and copper from rocks (Das et al., 1999; Hong and Valix, 2014). Among the advantages of biomining are its affordability and environmental sustainability. For instance, it can reduce metal contamination and improve the recycling of elements from electrical waste (Jerez, 2017), or reduce the use of environmentally damaging toxic compounds such as cyanides (Hilson and Monhemius, 2006). Although the use of acidophilic iron and sulfur-oxidisers is one well-developed method, heterotrophic microorganisms, including bacteria and fungi, are effective in bioleaching in environments with circumneutral and alkaline pH. One mechanism by which such organisms can biomine is through the release of chelating compounds that sequester the element of interest (Sukla and Panchanadikar, 1993; Bosecker, 1997; Rezza et al., 2001; Schippers et al., 2014; Reed et al., 2016).

Beyond Earth, the same processes could be applied on other planetary bodies, for example on the Moon or Mars (Cockell, 2010, 2011; Montague et al., 2012; Menezes et al., 2015; Jerez, 2017; Volger et al., 2020), allowing *in situ* resource utilization (ISRU). To successfully expand biomining beyond Earth, we need to gain knowledge of how altered gravity conditions, such as microgravity, lunar gravity and Martian gravity, change microbial interactions with minerals. We know that microgravity can influence growth and metabolic processes (Horneck et al., 2010; Moissl-Eichinger et al., 2016; Huang et al., 2018). The exact effects of microgravity on single cells and communities are a focus of research (Gasset et al., 1994; Kacena et al., 1999; Leys et al., 2004; Crabbé et al., 2013; Foster et al., 2014; Santomartino et al., 2020) and fractional gravity levels between microgravity and 1 × *g* have been shown to have differential effects on organisms such as flagellates (Häder et al., 1996) and plants (Kiss et al., 2012; Kiss, 2014). By allowing for thermal convection and sedimentation, gravity influences the mixing of microbial nutrients and waste, thereby affecting growth and metabolism of cells. Based on these considerations, we could expect that different gravity conditions would induce changes in microbial interactions with minerals and consequently bioleaching.

Another important application of microorganisms is in bioremediation (e.g., Allard and Neilson, 1997; Wang et al., 2012; Adams et al., 2015). In this process, microorganisms are used to sequester, degrade or immobilize toxic elements or compounds from soils and other substrates either to clean up anthropogenic pollution or transform natural environments that contain toxic levels of an undesired element or compound. One example is the use of bioremediation for the decontamination of wastewater and land from toxic and carcinogenic arsenic and its derivatives (Hunter, 2002; Kostal et al., 2004; Drewniak and Sklodowska, 2013). Bioremediation has huge economic implications, both for reducing the impact of human industry on the natural environment and as a means to produce a circular economy whereby human waste and pollution can be mitigated and even recycled into useful products (Gillespie and Philp, 2013), including radioisotopic materials (Brim et al., 2000; Lloyd and Renshaw, 2005). Natural regolith on other planetary bodies also contains potentially toxic elements and compounds, for example perchlorates on Mars (Davila et al., 2013). Microbes could be used to bioremediate soils and human waste on other planetary bodies to eliminate toxicity or recycle elements. Thus, both biomining and bioremediation represent two microbially mediated processes of potential benefit to the building of self-sustaining human settlements beyond Earth.

One element with strong industrial interest is vanadium, a metal that can be extracted from minerals and other materials using microorganisms (Mishra et al., 2007; Mirazimi et al., 2015; Dong et al., 2019) and can be immobilized by microbial reduction in bioremediation schemes (Mirazimi et al., 2015; Zhang et al., 2019a, b). This element is used in ferrous and non-ferrous (including nickel, titanium, aluminum, and chromium) alloys to enhance hardness, tensile strength and fatigue resistance (Moskalyk and Alfantazi, 2003). On Earth, these alloys find use in structural materials, rails, metallurgical tools, applications that beyond Earth could apply to the fabrication of rovers, pressurized buildings, and particularly objects exposed to lunar and Martian dust and its corrosive, abrasive properties (Ferguson et al., 1999; Stubbs et al., 2005). The global iron and steel industry accounts for about 85% of the use of vanadium on Earth. The element is also used in catalysis in a variety of industrial applications (Wachs, 2013), in the production of superconducting materials (Wexler and Corak, 1952; Bjergfelt et al., 2019) and in batteries (Baroch, 2013). The low neutron-absorbing capacity of vanadium makes it a useful additive in the fabrication of alloys for nuclear reactors (Loria, 1976), including fusion reactors (Smith et al., 1985). The high thermal conductivity and low thermal expansion coefficient of vanadium-base alloys makes them valuable in situations where low thermal stress-failure of materials is required. The metal has been used to improve bioremediation schemes or itself has been the focus of bioremediation where it exists at toxic levels (Gan et al., 2020; Shi et al., 2020; Wang et al., 2021). Vanadium also has roles in pharmaceutical applications (Rehder, 2015). World reserves of vanadium are expected to be sufficient for a century or more. Although it is not a critically limited element on Earth, as with all elements it would be beneficial to find ways to acquire it locally on other planetary bodies hosting a human presence to reduce the mass and energy costs of transportation into space. Interestingly, vanadium is found in carbonaceous chondrites, and its role in the origin of life has been investigated (Campitelli and Crucianelli, 2020). From a microbial evolutionary perspective, vanadium-dependent enzymes (nitrogenases and haloperoxidaes) have been reported (Garcia et al., 2020), and vanadate (an anionic coordination complex of vanadium) can be used as an electron acceptor in bacterial respiration (Rehder, 2015).

The European Space Agency (ESA) BioRock experiment was performed on the International Space Station (ISS) in 2019 to investigate the leaching of elements from basalt, an analog for regolith material on the Moon and Mars (Ruzicka et al., 2001; McSween et al., 2009; McMahon et al., 2013), by three species of heterotrophic microorganisms. On the ISS, the experiment compared bioleaching at three different levels of gravity: microgravity, simulated Mars and simulated terrestrial gravity. We report results on the bioleaching of vanadium, demonstrating the potential for vanadium biomining in the gravity regimes prevailing on asteroids and Mars. The data advance beyond our former results showing biomining of rare earth elements in space (Cockell et al., 2020), and demonstrate the general principles of the biological sequestration of metal ions in bioremediation and recycling efforts beyond Earth.

## Methods

### BioRock Experiment

BioRock was an experiment proposed to European Space Agency (ESA) in response to the International Life Science Research Announcement from 2009 (ILSRA-2009) seeking research with evidence of ground-based success and a high likelihood of successful completion. The project was selected as a candidate flight in 2010 and subsequent bioreactor hardware design has been described (Loudon et al., 2018). The 3-week experiment began on the International Space Station on July 30, 2019 and ended on August 20, 2019.

### Microorganisms and Growth Media

Three bacterial species were used to investigate bioleaching under different gravity regimens. To allow comparison between organisms, the following requirements had to be met by the microorganisms that were selected for this study: (1) ability to tolerate desiccation for the experiment preparation, (2) ability to grow on solid surfaces and/or form biofilms, (3) ability to interact with mineral surfaces and/or bioleach aerobically, and (4) ability to grow in identical medium under identical experimental conditions.

The microorganisms used were:

*Sphingomonas desiccabilis* CP1D (DSM 16792; Type strain), a Gram-negative, non-spore-forming, non-motile bacterium, which was isolated from soil crusts in the Colorado plateau (Reddy and Garcia-Pichel, 2007). The resistance of *S. desiccabilis* to simulated Martian brine has been studied, with a greater demonstrated resistance of biofilms compared to planktonic cells (Stevens et al., 2019). *Sphingomonas* spp. have previously been shown to be capable of bioleaching activity such as dissolving insoluble phosphate by chelation (Senoo et al., 1996, Teng et al., 2013). In bioleaching, *S. desiccabilis* was also demonstrated to preferentially leach heavy (Gd to Lu) rare earth elements (REEs) over light (La to Eu) REEs (Cockell et al., 2020).

*Bacillus subtilis* NCIB 3610 (DSM 10; Type strain), a Gram-positive, motile, spore- and biofilm-forming bacterium naturally found in a range of environments, including rocks (Song et al., 2007). The organism has been used in several space experiments (Kacena et al., 1999; Horneck et al., 2010).

*Cupriavidus metallidurans* CH34 (DSM 2839; Type strain), a Gram-negative, motile, non-spore forming bacterium. Strains of this species have been isolated from metal-contaminated and rock environments (Diels and Mergeay, 1990; Brim et al., 1999; Goris et al., 2001; Sahl et al., 2008; Kelly et al., 2011; Mijnendonckx et al., 2013). The organism has been previously used in space experiments (Leys et al., 2009).

The medium used for the BioRock experiment was R2A (Reasoner and Geldreich, 1985) at 50% v/v of the published component concentrations as it supported growth of all three microorganisms (Loudon et al., 2018), allowing for comparisons. The composition was (g/L): yeast extract, 0.25; peptone, 0.25; casamino acids, 0.25; glucose, 0.25; soluble starch, 0.25; Na-pyruvate, 0.15; K_2_HPO_4_, 0.15; MgSO_4_.7H_2_O, 0.025 at pH 7.2.

NOTOXhisto (Scientific Device Laboratory, IL, United States), a formalin-free fixative compatible with safety requirements on the International Space Station (ISS), was used to halt bacterial metabolism at the end of the experiment at a ratio of 1:5 v/v.

### Bioleaching Substrate

Basalt acted as the substrate for bioleaching. Its elemental composition, including vanadium content, was determined by ICP-MS (inductively coupled plasma mass spectrometry), and bulk composition was determined by X-ray Fluorescence (XRF). Composition of the rock was previously reported elsewhere (Cockell et al., 2020). Vanadium content is reported here. The chosen experimental substrate was an olivine basalt rock collected near Gufunes, Reykjavik in Iceland (64°08′22.18″N, 21°47′21.27″W), chosen for its chemical composition, which is similar to that of basalts found on the Moon and Mars with many siderophile and volatile lithophile elements (Ruzicka et al., 2001; McSween et al., 2009).

The rock was cut into slides of 1.5 cm × 1.6 cm and 3 mm thickness. The mean mass of 15 of these slides was 1.87 ± 0.06 g (mean ± standard deviation). The rock was not crushed, as might be the done in large-scale bioleaching to enhance the surface area accessible to the microbes, because the BioRock project was also concerned with quantifying the formation of microbial biofilms, which was optimal when performed with fluorescence microscopy on a contiguous mineral surface.

### Sample Preparation for Flight

The basalt rock slides were sterilized by dry-heat sterilization in a hot air oven (Carbolite Type 301, United Kingdom) for 4 h at 250°C. This treatment did not change the mineralogy of the rocks as determined by X-Ray Diffraction (XRD) before and after treatment.

Single strain cultures of each organism were desiccated on the slides as follows. All manipulations were performed using aseptic techniques in a laminar flow-hood.

#### Sphingomonas desiccabilis

An overnight culture of the strain was grown in 100% v/v R2A at 20–22°C until reaching stationary phase. Then, 1 mL of the culture was inoculated on each basalt slide (≈ 1 × 10^9^ cells per slide) and the samples were air-dried at room temperature (≈20–25°C).

#### Bacillus subtilis

Spores were produced as described previously (Fuchs et al., 2017). For each basalt slide, 10 μL of a ≈1 × 10^8^ spores/mL solution were used as inoculum, *i.e.*, 1 × 10^6^ spores per slide, and air-dried at room temperature (≈20–25°C).

#### Cupriavidus metallidurans

Samples were prepared using a freeze-dry protocol (Belgian Co-ordinated Collection of Micro-organisms, BCCM) involving a cryoprotectant as described in Santomartino et al. (2020).

For sterile controls, sterile basalt slides without cell inoculation were used. After preparation, all samples were stored at room temperature (20–25°C) until integration into the culture chambers within the bioreactor.

### Flight Experimental Setup

**FIGURE 1:**
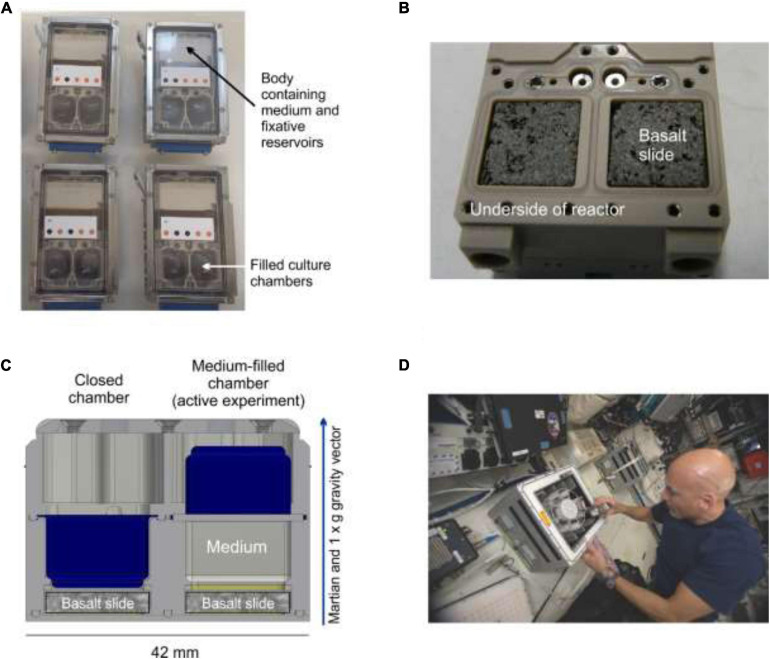
The BioRock Experimental Unit (EU). **(A)** Top-down image of four Experimental Containers (EC) containing one EU each, showing medium inflated culture chambers. **(B)** Rear side image of the EU showing two basalt slides inserted into the bottom of the culture chambers before closure of EU. **(C)** A lateral cross-section through the culture chamber showing the position of the basalt slide at the back of the chamber and the principle of medium injection and inversion of the membrane (in blue; left side closed, right side inflated with medium). A scale bar shows the size of the unit, which also applies to the images in **(A)** and **(B)**. **(D)** ESA Astronaut Luca Parmitano inserts an EC into a KUBIK incubator on board the ISS (credit: ESA).

The hardware design, assembly and filling procedure has been described previously (Loudon et al., 2018). Each Experiment Unit (EU) of the BioRock apparatus was designed to accommodate two independent basalt slides in two independent sample chambers (Figure 1). Each EU contained culture medium and fixative reservoirs (Figure 1A). The immobilization and bioleaching of vanadium was investigated in a context of aerobic microbial growth, thus it was important for the culture chambers to allow oxygen diffusion. To allow oxygen diffusion without contaminating the cultures, each chamber was equipped with a deformable, gas-permeable, silicone membrane (Figures 1B,C) (Loudon et al., 2018). After integration of the basalt slides, the medium and fixative reservoirs were filled with 5 mL of medium and 1 mL of fixative for each sample, respectively. The culture chambers and surrounding ducts were purged with ultrapure sterile N_2_ gas.

All the samples were integrated into the EUs using strict aseptic procedures at the NASA Kennedy Space Center. In total 36 samples were mounted in 18 EUs for the flight experiment, and 12 samples were mounted in 6 EUs for the reference ground experiment. All samples in all the gravity conditions were present in triplicate. The EUs were integrated into a secondary container, called the Experiment Container (EC) that added an extra level of containment required for the fixative (Figure 1A).

After integration, 18 flight ECs were stored at room temperature (≈20–25°C) for two days. The ECs were launched to the ISS on board a SpaceX Dragon capsule, Falcon-9 rocket as part of CRS-18 (Commercial Resupply Services) mission on July 25, 2019 from the NASA Kennedy Space Center, Cape Canaveral, Florida. On arrival at the ISS, ECs were stored on-board at 2.1°C in the Minus Eighty-Degree Laboratory Freezer for ISS (MELFI), which contains a refrigeration compartment.

On the day of the start of the experiment (July 30, 2019), the ECs were installed into two KUBIK incubators by astronaut Luca Parmitano, pre-conditioned to a temperature of 20°C (Figure 1D). Medium injection was performed autonomously, triggered by internal clocks within the ECs, after they were powered by the KUBIK incubator. Thereafter, the astronaut removed the ECs and took photographs of all culture chambers that were visible through the window in the hardware to document proper medium supply and to define the baseline for later comparison with the same chamber at the end of the active experimental growth phase. After image acquisition, the ECs were reinstalled into the two KUBIKs. Each KUBIK incubator contains a centrifuge. The centrifuge of the first KUBIK facility was set to produce Earth gravity (1 × *g* = 9.81 m/s^2^) at the surface of the basalt slide where the bioleaching is occurring, while the centrifuge of the second KUBIK was set to provide approximately Mars gravity (0.4 × *g* = 3.71 m/s^2^. Mars gravity is strictly 0.38 × *g*, but finer *g* resolution is not possible to set in the KUBIK) at the surface of basalt slide. The gravity vector points away from the basalt rock. This ensures that cells growing on the rock surface are forming true biofilm and not the result of gravitational sedimentation onto the rock (the BioRock experiment was also focused on studying biofilm growth on rock). This approach is referred to as the Calgary method of biofilm analysis (Ceri et al., 1999). Gravity levels were measured using a miniature digital accelerometer (ADXL313, Analog Devices) mounted on a Printed Circuit Board (PCB) fixed to the bottom of the EC. The distance between the top face of the basalt slide and the plane of the top face of the PCB was 10.3 mm. A correction factor was applied to account for the longer rotation radius at the basalt slide (Santomartino et al., 2020). The microgravity-exposed ECs were divided equally between both KUBIKs and inserted in the static slots of the facility. The experiment was conducted for 21 days.

To stop the cultures from growing, NOTOXhisto as fixative was injected automatically into the culture chambers on August 20, 2019. The samples were removed from the KUBIK incubators and images of the culture chambers were taken. The ECs were refrigerated at 2.1°C until return to Earth.

The temperature of the ECs from pre-flight until postflight was measured using temperature loggers (Signatrol SL52T sensors, Signatrol, United Kingdom) on the rear of four of the 18 ECs, which were interrogated on Earth after the experiment. These data showed that temperatures did not exceed 7.1°C from pre-flight hand-over until storage after arrival at the ISS. During on-board storage, both before and after the 21-day period of culturing, temperatures were constant at 2.1°C. During culturing, the loggers recorded a temperature of 20.16°C in both KUBIKs. The ECs were downloaded from the ISS, packed in a ‘double coldbag’ provided by NASA. Splashdown occurred in the Pacific Ocean on August 27 and handover of the ECs to the investigators occurred on August 29 at Long Beach Airport, LA, United States. Between removal from storage on August 26 on ISS and hand-over to the science team on August 29, the temperature loggers recorded a temperature of 6.6°C, rising transiently to 7.1°C. The ECs were stored in a refrigerated insulated box and transferred to the NASA Ames Research Centre for sample removal on August 30.

### Ground Experiment

Parallel to the space experiments, a 1 × *g* ground experiment (true Earth gravity) was run for comparison with simulated Earth gravity samples on board the ISS. Six ECs for the ground experiment were shipped from the NASA Kennedy Space Center to the NASA Ames Research Centre under cooled (4°C) conditions. For the experiment, the six ECs were attached to a power system (KUBIK Interface simulation station, KISS) with leads running from the supply into a 20°C laboratory incubator (Percival E30BHO incubator). The ground reference experiment was started two days after the space experiment and the procedure for the space experiment was replicated: medium injection, first image acquisition, 21-day experiment, fixation, second image acquisition, and cold storage at 4°C. The temperatures of the ECs measured by temperature logger (see above) on two of the ECs were 3.6 and 4.5°C, respectively, during shipment to NASA Ames. During the 21 days of the main experimental phase, the loggers recorded a temperature of 20.6°C. During post-experiment storage, the temperature was 3.1°C.

### Sample Recovery

Liquid and basalt-slide removal from the ECs was performed at the NASA Ames Research Center. From the 6 mL of total bulk fluid per EC, an aliquot of 3 mL was taken and 65% (v/v) nitric acid was added to a final concentration of 4% (v/v) to fix ions and minimize attachment and loss to walls of the container in order to gain a more accurate understanding of the bioleaching efficacy. These samples were stored at 4°C until further analysis.

NOTOXhisto fixative injection was successful for all the ECs on the ISS. However, it failed in four of the twelve ground experimental chambers: one *B. subtilis* chamber, two *C. metallidurans* chamber and one sterile control sample. In these cases, 1 mL of NOTOXhisto was added to the liquid samples before the liquid preparation procedures were carried out.

Two culture chambers out of 48 (36 flight + 12 ground) were observed to have contamination: an ISS sterile control chamber in microgravity, juxtaposed to a *B. subtilis* chamber, was contaminated with cells that were morphologically identical to *B. subtilis*. In the ground control samples, a sterile chamber, juxtaposed to a *B. subtilis* chamber, had a cellular contaminant at low concentration that formed a white pellet on centrifugation that was morphologically different from *B. subtilis*. NOTOXhisto fixation prevented successful DNA extraction and identification in both cases. These data points were removed from the calculations.

All samples were shipped back to the University of Edinburgh under thermal control (4°C) by Altec SpA (Torino, Italy).

### ICP-MS Analysis of Samples

Upon return to Edinburgh, United Kingdom, the 3 mL of acid-fixed sample were prepared as follows: each sample was split into three one-mL batches in 1.5 mL tubes and centrifuged at 10,000 × *g* (IEC MicroCL 17 centrifuge, Thermo Scientific) for ten minutes to pellet cells and cell debris. The supernatant was collected into a 15 mL tube and analyzed by ICP-MS to determine the bulk fluid vanadium concentrations. Cell debris pellets were washed twice in ddH_2_O and the wash solution was pooled from the three samples. Nitric acid was added to the pooled fluid to a final concentration of 4% (v/v) to prevent binding of ions to the walls of the tubes, and the samples were analyzed by ICP-MS. This sample determined the quantity of vanadium that was washed off the cell matter. To determine the residual amount of vanadium that was present in the cell matter, the cell pellet was transferred to an acid-washed glass serum vial pre-baked at 450°C in an oven (Carbolite Type 301, United Kingdom) for four hours to remove organic molecules. The vial containing the pellet was heated at 450°C for four hours to remove organic carbon. After cooling, 1.5 mL of ddH_2_O containing 4% (v/v) of nitric acid was added and the samples were analyzed by ICP-MS.

ICP-MS analysis was carried out as described above on the R2A 50% (v/v), NOTOXhisto and ddH_2_O samples. We did not examine the cryoprotectant for *C. metallidurans*. However, as we did not observe significant vanadium enhancement in the experiments, we conclude that the protectant did not contain additional vanadium.

All samples were analyzed by ICP-MS using an Agilent 7500ce (with octopole reaction system), employing an RF forward power of 1540 W and reflected power of 1 W, with argon gas flows of 0.81 L/min and 0.20 L/min for carrier and makeup flows, respectively. Sample solutions were taken up into the micro mist nebuliser using a peristaltic pump at a rate of approximately 1.2 mL/min. Skimmer and sample cones were made of nickel.

The instrument was operated in spectrum-multi-tune-acquisition-mode and three runs per sample were employed. Vanadium was analyzed in the helium mode to minimize overlap with other isotopes.

NIST standard reference materials were employed as standards to calibrate the instrument: SLRS-4 (concentration of vanadium, 0.32 ppb) and SRM1640a (concentration, 14.93 ppb). The detection limit for vanadium was 0.1 ppb.

Raw ICP-MS data (determined in μg/L) was used to obtain the absolute quantity of vanadium in the culture chamber and took into account dilution factors applied during ICP-MS analysis.

### Vanadium Concentration in Basalt Substrate

Vanadium concentrations in the basalt slide were determined by ICP-MS. Three separate basalt slices were crushed under aseptic conditions in a mortar and pestle. Between 25 and 50 mg of homogenized basalt dust were transferred to Savillex Teflon vessels. Rock standards (basalt standards BIR-1, BE-N, BCR-2, BHVO-1) were prepared in the same way. Two blanks were included (*i.e.*, sample without basalt). Three milliliters of double distilled HNO_3_, 2 mL HCl and 0.5 mL HF were added to each vessel. HF was added after the other acids to prevent disassociation, formation and precipitation of aluminum fluorides. Samples were placed on a hot plate for digestion overnight (temperature of 100−120°C) and checked for complete dissolution. Samples were evaporated on the hot plate to dryness. Five milliliters of 1 M HNO_3_ were added to each vessel. Lids were added and the samples returned to the hotplate for a second dissolution step. Samples were further diluted with 2–5% nitric acid (v/v) for ICP-MS analysis.

Analysis was carried out on a high resolution, sector field, ICP-MS (Nu AttoM). The ICP-MS measurements of vanadium were performed in low resolution (300), in Deflector jump mode with a dwell time of 1 ms and 3 cycles of 500 sweeps. Data were reported in micrograms vanadium per gram basalt.

### Statistical Analysis

Analysis of the leaching data was performed at several levels of granularity using SPSS Statistics (IBM). One- and two-way ANOVAs were used to assess significant differences between gravity conditions, organisms, ground and space samples, and between controls, in combinations described in the results. Tukey tests were performed where appropriate to examine pairwise comparisons. To investigate differences between gravity conditions and organisms or controls a two-sample independent Student’s *t*-test was used between pairs of conditions. Tests for normality of data and equal variances (Levene’s tests) were carried out. The number of samples was *n* = 3 for all conditions, with the exception of sterile controls in microgravity and the ground experiment (true Earth gravity), in which *n* = 2 due to contamination.

## Results

### Vanadium in Basalt Substrate

The vanadium concentration in the basalt was 175.03 ± 21.01 ppm (mean ± standard deviation), which is within the range of concentrations found in some lunar basalts (e.g., low Ti-basalts; 106-227 ppm; Hopkins et al., 2019, and Apollo 12 lunar samples; 126-296 ppm; Lipschutz et al., 1971) and Martian basalts (e.g., between 100 and 350 ppm; Herd et al., 2002; Treiman, 2003). These values are higher than those typically found in meteorites (ordinary, carbonaceous, and enstatite chondrites), which range from 41–84 ppm (Nichiporuk and Bingham, 1970; McDonough and Sun, 1996). The oxidation state of vanadium in basaltic substrates varies, but generally it is in the 3 + or 4 + state in surface basalts on Earth (Righter et al., 2006).

### Vanadium Mining Can Be Microbially Enhanced in Space

**FIGURE 2:**
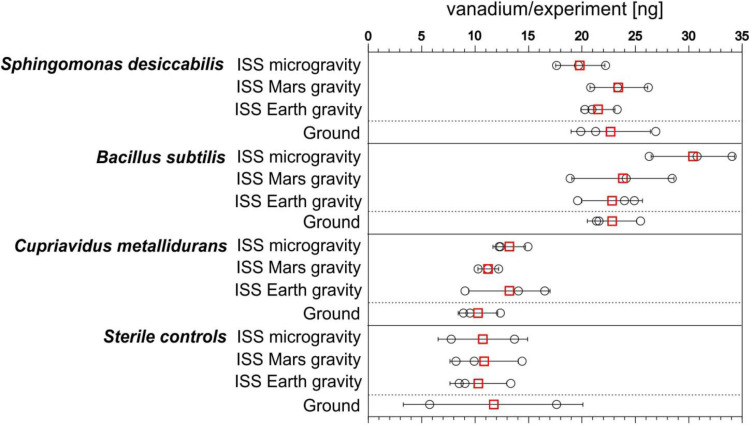
Bioleaching and control leaching of vanadium from basalt on the International Space Station (ISS) and on Earth. Total quantities (ng) of vanadium in each of the experimental flight and ground control samples at the end of the experiment for each of the three organisms examined and sterile samples. ○ shows single measurements and the mean is given as red □. Error bars represent standard deviations. *n* = 3, except for sterile controls in microgravity and the ground experiment, in which *n* = 2 due to contamination. Data in Supplementary Table 1.

Mean and standard deviations of the absolute quantities of vanadium in the chamber fluid (6 mL) were calculated. The quantity of vanadium in the medium and fixative (8.35 ± 0.05 ng, mean ± standard deviation) were subtracted (Figure 2 and Table 1). In the space station biological experiments (labeled ISS in Figure 2), across all three gravity conditions, mean values for *S. desiccabilis* and *B. subtilis* ranged from 19.84 ± 2.29 to 30.38 ± 3.90 ng (mean ± standard deviation). For *C. metallidurans* quantities ranged from 11.23 ± 0.96 to 13.21 ± 3.80 ng. In sterile samples on ISS, vanadium quantities ranged from 10.30 ± 2.65 to 10.84 ± 3.19 ng. In the ground experiment, the mean values of vanadium measured for the biological experiments for *S. desiccabilis* and *B. subtilis* were 22.69 ± 3.72 and 22.81 ± 2.31 ng, respectively. The value for *C. metallidurans* was 10.26 ± 1.85 ng. A value of 11.69 ± 8.40 ng was measured for the ground sterile control.

**TABLE 1:** Data associated with the biomining of vanadium.

		1	2	3	4
		Quantity (ng)	Comparison with control (%)	Comparison with quantity in basalt (%) x 10^–4^	Percentage of total leached in cell pellet (%)
*S. desiccabilis*	Microgravity	19.84 ± 2.29	184.92 ± 75.33	60.61 ± 7.01	1.39 ± 0.51
	Mars gravity	23.45 ± 2.70	216.32 ± 68.43	71.65 ± 8.25	1.69 ± 0.40
	Earth gravity	21.50 ± 1.59	208.70 ± 55.85	65.70 ± 4.85	0.96 ± 0.23
*B. subtilis*	Microgravity	30.38 ± 3.90	283.22 ± 116.44	92.82 ± 11.91	0.96 ± 0.36
	Mars gravity	23.83 ± 4.78	219.78 ± 78.37	72.805 ± 14.62	1.86 ± 0.53
	Earth gravity	22.83 ± 2.84	221.59 ± 63.30	69.74 ± 8.66	1.74 ± 0.85
*C. metallidurans*	Microgravity	13.19 ± 1.53	122.97 ± 50.10	40.30 ± 4.66	2.67 ± 1.05
	Mars gravity	11.23 ± 0.96	103.54 ± 31.78	34.30 ± 2.94	4.20 ± 1.73
	Earth gravity	13.21 ± 3.80	128.21 ± 49.47	40.35 ± 11.61	1.67 ± 0.35
Sterile ISS control	Microgravity	10.73 ± 4.19	−	32.77 ± 12.80	−
	Mars gravity	10.84 ± 3.19	−	33.12 ± 9.76	−
	Earth gravity	10.30 ± 2.65	−	31.47 ± 8.09	−
Ground 1 *g* experiment	*S. desiccabilis*	22.69 ± 3.72	194.13 ± 143.11	69.33 ± 11.36	2.46 ± 1.31
	*B. subtilis*	22.81 ± 2.31	195.12 ± 141.63	69.68 ± 7.07	1.41 ± 0.31
	*C. metallidurans*	10.26 ± 1.85	87.78 ± 65.04	31.35 ± 5.65	2.73 ± 1.06
	Sterile control	11.69 ± 8.40	−	35.72 ± 25.67	−

*Columns: (1) Mean (± standard deviation) of quantities of vanadium (ng) in the chamber fluid (6 mL) at the end of the experiment (50% R2A and fixative contribution subtracted). Data presented in Figure 2; (2) Mean (± standard deviation) leached quantity of vanadium in biological experiments expressed as a percentage of sterile controls; (3) Quantity of vanadium leached in biological experiments as percentage of total quantity in basalt slide; (4) Total yield of vanadium absorbed by cell pellet, expressed as a percentage of total yield in the pellet and bulk chamber fluid (%). Mars gravity and Earth gravity refer to simulated Martian (0.4 × *g*) and terrestrial (1 × *g*) gravity on ISS.*

**FIGURE 3:**
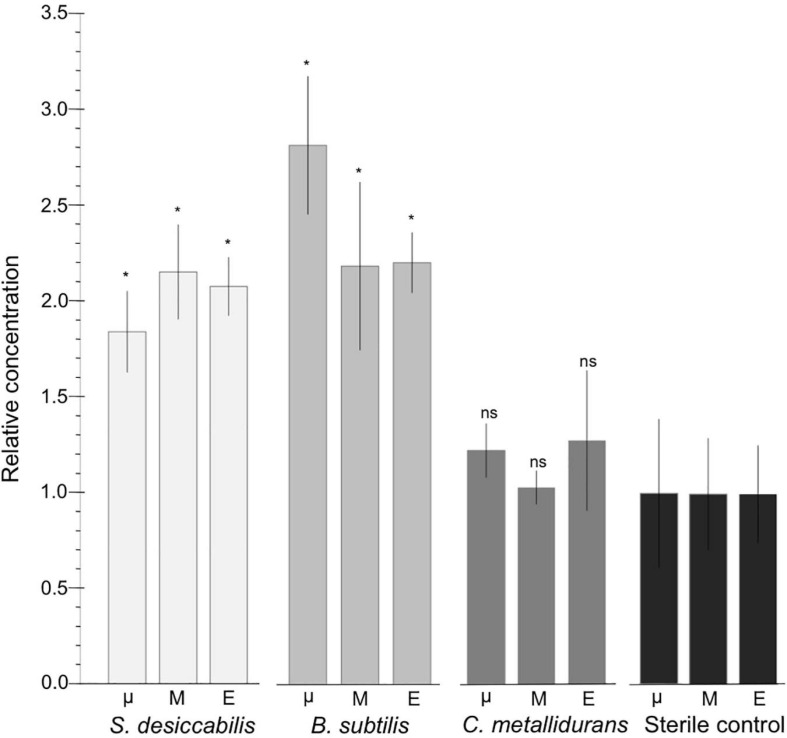
Biological enhancement of vanadium leaching on ISS. Graph showing relative quantity of vanadium leached into the culture chamber for the different gravity conditions and organisms on ISS (μ = microgravity; M = Mars gravity; E = Earth gravity). Data shown as a ratio of biology versus sterile controls. Asterisks reflect samples that are significantly different from the sterile controls (*p* < 0.05). ‘ns’ means not significant. Sterile control data are also shown. *n* = 3, except for sterile controls in microgravity in which *n* = 2 due to contamination.

On the ISS, mean quantities of vanadium in the biological experiments for *S. desiccabilis* and *B. subtilis* were in all cases higher than the quantity in the corresponding sterile controls, ranging from 184.92 ± 75.33 to 283.22 ± 116.44% of the sterile control. For *C. metallidurans* values ranged from 103.54 ± 31.78 to 128.21 ± 49.47% (shown as ratios in Figure 3). In the ground-based experiments, the values for *S. desiccabilis* and *B. subtilis* were 194.13 ± 143.11 to 195.12 ± 141.63% of the sterile control value. The value for *C. metallidurans* was 87.78 ± 65.04% of the sterile control value (Table 1).

Statistical analysis was carried out to investigate whether the presence of the bacteria increased vanadium extraction compared to sterile controls. One-way ANOVAs were used to assess the difference between biological samples and the sterile controls in all three gravity conditions (microgravity, simulated Mars, and simulated Earth gravity) on board the ISS. For *S. desiccabilis* the leaching of vanadium in the biological experiments was found to be significantly higher than in the controls across all gravity conditions (ANOVA: *F*(1,15) = 74.95, *p* < 0.001). For *B. subtilis*, biological leaching also produced significantly higher quantities of vanadium compared to sterile controls across all three gravity conditions (ANOVA: *F*(1,15) = 58.69, *p* < 0.001). For *C. metallidurans*, biological leaching did not produce significantly higher quantities of vanadium compared to sterile controls across all three gravity conditions (ANOVA: *F*(1,15) = 2.48, *p* = 0.136).

Student’s *t*-test was used to investigate the effect of individual gravity conditions and ascertain the statistical significance of bioleaching under gravitational conditions (Table 2). For both *S. desiccabilis* and *B. subtilis*, the quantity of vanadium leached from the basalt was statistically higher than sterile controls (*p* < 0.05) in all gravity conditions on the ISS. In the ground-based (true Earth gravity) experiment, differences between the biological experiments and sterile controls were not significant (*S. desiccabilis* vs. control, *p* = 0.126; *B. subtilis* vs. control, *p* = 0.101). Comparisons for *C. metallidurans* between the biological experiments and sterile controls in different gravity conditions did not show significant differences.

**TABLE 2:** Student’s *t*-test comparisons (*p*-values) between vanadium leaching in selected treatments.

*S. desiccabilis on ISS*	
Microgravity (biology vs. control)	0.047*
Mars gravity (biology vs. control)	0.006*
Earth gravity (biology vs. control)	0.003*
Microgravity biology vs. Mars gravity biology	0.152
Microgravity biology vs. Earth gravity biology	0.361
Mars gravity biology vs. Earth gravity biology	0.341
***B. subtilis on ISS***	
Microgravity (biology vs. control)	0.013*
Mars gravity (biology vs. control)	0.017*
Earth gravity (biology vs. control)	0.005*
Microgravity biology vs. Mars gravity biology	0.140
Microgravity biology vs. Earth gravity biology	0.053
Mars gravity biology vs. Earth gravity biology	0.771
***C. metallidurans on ISS***	
Microgravity (biology vs. control)	0.394
Mars gravity (biology vs. control)	0.852
Earth gravity (biology vs. control)	0.338
Microgravity biology vs. Mars gravity biology	0.132
Microgravity biology vs. Earth gravity biology	0.995
Mars gravity biology vs. Earth gravity biology	0.431
**ISS control comparisons**	
Microgravity control vs. Mars gravity control	0.895
Microgravity control vs. Earth gravity control	0.974
Mars gravity control vs. Earth gravity control	0.833
**Ground 1×g experiments**	
*S. desiccabilis* vs. control	0.126
*B. subtilis* vs. control	0.101
*C. metallidurans* vs. control	0.778
**Ground and ISS comparisons**	
*S. desiccabilis* ground vs. ISS Earth gravity	0.635
*B. subtilis* ground vs. ISS Earth gravity	0.994
*C. metallidurans* ground vs. ISS Earth gravity	0.294
Ground control vs. ISS Earth gravity control	0.793

p-values below 0.05 are shown as *. ‘Control’ refers to sterile control.

### Final Quantities of Vanadium Were Not Influenced by Gravity Condition

Statistical analysis was carried out to investigate whether there were significant differences in the final quantity of vanadium under the four gravity conditions: microgravity, simulated Mars, and simulated Earth gravity on board the ISS, and ground (true Earth) gravity in the biological leaching experiments. There was no significant difference among the four gravity conditions for any of the three microorganisms (*S. dessicabilis*: ANOVA: *F*(3,8) = 1.032, *p* = 0.42; *B. subtilis*: ANOVA: *F*(3,8) = 3.10, *p* = 0.089; *C. metallidurans*: ANOVA: *F*(3,8) = 1.23, *p* = 0.360) and *post hoc* Tukey tests revealed no significant differences in any pairwise comparisons for the three organisms.

Statistical analysis (one-way ANOVA) was carried out to investigate whether there were significant differences among the final quantities of vanadium in the sterile controls in the four gravity conditions: microgravity, simulated Mars, and simulated Earth gravity on board the ISS, and ground (true Earth) gravity. There was no significant difference among the four gravity conditions (ANOVA: *F*(3,6) = 0.038, *p* = 0.989) and *post hoc* Tukey tests revealed no significant differences in any pairwise comparisons.

Student’s *t*-test results for pairwise comparisons between the biological conditions and between the sterile controls on ISS were also undertaken and are shown in Table 2. No significant differences were observed (*p* < 0.05) in any pairwise comparison.

### Performance of Biomining in Space Compared to Ground (True Earth Gravity) Conditions and Ground Experiment

For the three organisms, there was no significant difference (*S. desiccabilis*, *p* = 0.635; *B. subtilis*, *p* = 0.994, *C. metallidurans*, *p* = 0.793) between the simulated Earth gravity biological experiment on the ISS and the ground-based (true Earth gravity) biological experiment (Table 2). Student’s *t*-tests showed that there was no significant difference in the sterile controls between leaching in simulated Earth gravity on the ISS and in the sterile ground-based (true Earth gravity) experiment (*p* = 0.793) consistent with ANOVA results reported above.

### Total Quantity of Vanadium Leached as a Proportion of the Rock Content

The total mean quantity of vanadium elements released as a percentage of the available quantity in the rock in the different conditions is shown in Table 1. The highest mean percentage was leached in the *B. subtilis* cultures in microgravity (9.28 ± 1.19 × 10^–3^%). Across all bioleaching experiments on the ISS, mean percentages ranged from 3.43 ± 0.29 × 10^–3^% up to 9.28 ± 1.19 × 10^–3%^. In the ground-based (true Earth gravity) experiments mean values in the bioleaching experiments ranged from 3.14 ± 0.56 × 10^–3^% to 6.97 ± 0.71 × 10^–3^%. In the sterile control experiments, mean percentages leached from the basalt were comparable under the three gravity conditions, ranging from 3.15 ± 0.81 to 3.31 ± 0.98 × 10^–3%^. In the ground-based (true Earth gravity) sterile control mean percentage was 3.57 ± 2.57 × 10^–3%^.

### Quantities of Vanadium Attached to Cell Pellet

To test whether vanadium was absorbed by the cells, ICP-MS analyses of the cell pellets were performed (Table 1). The quantities of vanadium in all samples accounted for less than 5% of the total quantity leached into the growth chamber. Mean values across all conditions on the ISS and the ground ranged from 0.96 ± 0.36 to 4.20 ± 1.73%.

### Biomining Occurred Under Mildly Alkaline pH Conditions in the Space and Ground Experiment

pH is an important controlling factor in biomining. The NOTOXhisto fixative halts bacterial metabolism at the end of the 21 day experiment and lowers the final pH of the solutions. Ground experiments showed that the pH was circumneutral in the sterile control throughout the experiment (7.35 ± 0.04), but the presence of bacteria caused the pH to rise to 8.66 ± 0.01. There was a drop of 4 to 5 pH units after addition of the fixative (Cockell et al., 2020) and in all solutions, the final pH ranged between 4.16 ± 0.20 and 6.12 ± 0.01.

## Discussion

This study investigated the use of microorganisms to extract elements from basalt rock, an abundant regolith material found on the Moon and Mars (Ruzicka et al., 2001; McSween et al., 2009; McMahon et al., 2013), under microgravity, simulated Mars and simulated Earth gravities on the ISS. We investigated microgravity (with a *g* usually ≤ 10^–2^ × *g*) as the lowest gravity level possible in order to explore the effects of a lack of sedimentation on bioleaching, to understand the role of gravity in microbe-mineral interactions in general, and to gain insights into the plausibility of industrial biomining on asteroids and other low gravity planetary objects, in particular. A true Earth gravity ground experiment was also performed in parallel as a reference. As lunar gravity (0.16 × *g*) lies between microgravity and Mars gravity, a lack of a significant effect in these two latter gravity conditions would likely imply that lunar gravity would also have no effect. However, gravity conditions between microgravity and Earth gravity are not necessarily linear and so care should be taken in extrapolating microgravity and Mars gravity results to lunar gravity conditions (Häder et al., 1996; Kiss et al., 2012; Kiss, 2014).

Cells of the species *Sphingomonas desiccabilis* and *Bacillus subtilis* significantly enhanced the leaching of vanadium in all gravity conditions compared to the corresponding sterile microbe-free controls in the same gravity condition, demonstrating the potential of vanadium biomining under gravitational conditions associated with asteroids and Mars. The bacteria could achieve generally a two-fold increase of vanadium leaching compared to sterile controls.

The lack of a significant difference in the final quantities of leached vanadium among the different gravity conditions might seem unexpected because microgravity has previously been reported to influence microbial processes such as growth and biofilm formation (McLean et al., 2001; Kim et al., 2013). However, the final cell concentration in different gravity conditions in our experiment did not differ among gravity conditions for the three microorganisms, possibly indicating consistent growth of microorganisms regardless of gravity conditions (Santomartino et al., 2020). One explanation could be that even if gravity did affect cell growth rates in the lag or log phase, the bacterial cultures used the nutrient supply to eventually reach similar maximum cell concentrations, regardless of gravity, within about 72 h, the time generally required to achieve stationary phase. Almost identical final cell concentrations might then lead to similar leaching efficiencies and vanadium quantities among the different gravity conditions over the remaining period of the experiment. Such a hypothesis may also provide an explanation for the observation that the motility of the organisms (*S. desiccabilis* is non-motile and *B. subtilis* is motile) did not have an evident influence on the results (Santomartino et al., 2020).

Similar results for the bioleaching of rare earth elements by *S. desiccabilis* were observed from the same BioRock experiment (Cockell et al., 2020). No significant effect of the gravity condition was observed, although some single REEs showed a statistically significant difference among gravity conditions for *S. dessicabilis*. In these experiments, Er leaching was enhanced by 429.2 ± 92.0% on the ISS, while Yb leaching was increased by 767.4 ± 482.4% on the ground compared to non-biological controls (Cockell et al., 2020).

One difference between the results reported here and rare earth element leaching results (Cockell et al., 2020) is that in the latter case we did not measure a statistically significant difference between leaching in the biological microgravity experiment and its corresponding sterile control even though mean leaching was greater and comparable to other gravity conditions. This may have been caused by the loss of one of the microgravity sterile controls causing a greater standard deviation for the two remaining samples compared to the sterile controls in the other gravity conditions. However, the standard deviation of the vanadium quantity in the microgravity sterile controls was not higher than the other sterile controls. Although this may suggest that the effects of gravity on microbial extraction in microgravity depends on the element, the statistical analysis performed do not support any effect of gravity on microbial bioleaching.

The mechanism for vanadium bioleaching is not known. Unlike acidic bioleaching, our experiment operated at mildly alkaline conditions (Cockell et al., 2020), which are optimum for some heterotrophic organisms including bacteria and fungi, although we cannot exclude local reductions in pH around individual cells within the microenvironment of a microbial community that could be species specific. Vanadium ions are known to be strongly chelated by a wide variety of enzymes and other biomolecules (Crans et al., 1989). *Sphingomonas desiccabilis* produces extracellular polymeric substances (EPS), compounds that are known to enhance bioleaching in other organisms by complexing ions in EPS moieties such as uronic acid (Pogliani and Donati, 1999; Welch et al., 1999). One possible hypothesis is that EPS chelates vanadium ions, and removes them from solution. EPS has been implicated previously in vanadium ion binding in bioremediation schemes (Zhang et al., 2019a). Another factor may be enhanced leaching caused by direct microbe-mineral interactions. *S. desiccabilis* is capable of forming biofilms on the surfaces of the basalt, which could have enhanced cell-mineral interactions and thus the leaching of the vanadium from the rock. We did not study the elemental composition of the biofilms because we wished to study their growth patterns non-destructively, therefore we could not explore their role in vanadium bioleaching.

The specific mechanism behind enhanced vanadium bioleaching in *B. subtilis* is also unknown. Interestingly, we did not observe enhanced leaching of other elements by this organism in the same experiment (Cockell et al., 2020). Recently, Kucuker et al. (2019) showed that *B. subtilis* was not able to extract tantalum from capacitors. However, the organism has previously been shown to enhance the bioleaching of copper and nickel by producing a lipoprotein that binds to the copper or nickel in the supernatant (Rozas et al., 2017; Giese et al., 2019) and the binding of rare earth elements to the cell wall (Takahashi et al., 2005) suggests cell wall-binding as one mechanism of leaching.

Our results are consistent with other observations. Some *Sphingomonas* and *Bacillus* species have been described as vanadium-reducing related bacteria (VRB) and found as constituents of vanadium-polluted soils (Wang et al., 2020). We note that immobilization and sequestration of vanadium in our experiment, which was aerobic, is different from microbial metabolic reduction of the metal (He et al., 2021), but nevertheless these results are consistent with the role of *Sphingomonas* and *Bacillus* in vanadium cycling.

*Cupriavidus metallidurans* did not enhance leaching of vanadium. In a three-month preparatory phase for the BioRock experiments, the leaching of elements from crushed basalt by this organism on the Russian Foton-M4 capsule was investigated (Byloos et al., 2017). In this experiment, *C. metallidurans* demonstrated enhanced copper ion release, but other rock elements did not show significantly enhanced leaching. Although the microorganism was suspended in mineral water instead of organic medium, the results are consistent with those reported here.

Our experiment showed that the leaching capacities of microorganisms on Earth (Willscher and Bosecker, 2003; Lapanje et al., 2011) were replicated in different gravity conditions in space. Thus, Earth-based experiments provide insights into the biomining capacities of specific organisms in space and how they might be applied to extraterrestrial biomining operations.

After fixation, bioleaching could have continued as the fixative had reduced the pH of the solutions and low pH in itself enhances leaching. However, during storage, the temperature was maintained at 2.1°C on the ISS and below 7.1°C during sample return to minimize leaching activity, since low temperatures are known to reduce leaching rates as a consequence of generally lower chemical reaction rates at low temperatures (White et al., 1999). A reduction of the pH occurred in the sterile samples to similar values which were also treated with the fixative, allowing us to exclude the possibility that the lowered pH after fixation contributed to the enhanced microbial leaching when these values were compared to the sterile controls. Moreover, although we did not observe enhanced vanadium associated with the cell pellets, the reduced pH caused by fixation and during sample preparation may have released vanadium bound to cell surfaces.

In contrast to the experiment on the ISS, we did not observe a significant difference in vanadium leaching between the biological experiment and sterile controls for each organism in the ground experiment. We attribute this difference to be caused by the higher standard deviation in the ground sterile controls. This interpretation is consistent with the observation that the mean quantities of vanadium in the ground biological experiments in *S. desiccabilis* and *B. subtilis* are higher than the sterile controls and the absolute quantities in the biological experiments and the sterile controls for these organisms are comparable to those obtained on the ISS.

Comparing the simulated Earth gravity biological experiments on the ISS with the ground-based biological experiments (true 1 × *g* control), we found no significant difference in each of three organisms. Furthermore, no significant difference was observed between the ISS and ground sterile controls. These data suggest that the Earth gravity samples on the ISS can be a realistic simulation of true Earth gravity controls, but our previous data show that differences can occur in some types of microbial measurements such as final cell concentrations (Santomartino et al., 2020) and also bioleaching efficiency (Cockell et al., 2020), and differences between space simulated 1 × *g* and true 1 × *g* have been observed in plants for instance (Guisinger and Kiss, 1999).

Our experiment was not a test of an optimized commercial bioleaching operation on other planetary bodies. The basalt rock was not crushed because we wanted to quantify biofilm formation, which is easier on a flat contiguous rock surface, another goal of the BioRock experiment. This likely reduced the efficiency of vanadium bioleaching from the rock compared to the quantities that would be leached from crushed materials. Stirring was absent in the reactors as we wished to investigate the effects of microgravity and Mars gravity on cell growth in the absence of artificial mixing. Therefore, we assume that the yields of vanadium sequestration could be greatly enhanced by optimizing rock preparation and processing steps in a commercial mining or bioremediation/recycling process. In the case of biomining specifically, the mineral and elemental prospecting of the Moon and other planetary bodies might lead to the identification of rocks with higher vanadium concentrations than those we examined in this study, further improving mining yields. However, high vanadium concentrations can potentially lead to cell toxicity (Kamika and Momba, 2014). In our study, we did not investigate the effects of changing temperature conditions, but temperature is another factor that could be explored to optimize biomining yields.

Finally, our results demonstrate the principle of bioremediation and elemental recycling in space. Although vanadium toxicity is not an expected problem in any known regolith material in space, these experiments show the principle of using microorganisms and their exudates to bind to ions and sequester them from local planetary materials either to reduce their concentrations in polluted or naturally contaminated materials or recover elements from waste for downstream recycling.

In conclusion, the results demonstrate the potential for biological vanadium sequestration under Martian and asteroidal low gravity conditions with applications to biomining, bioremediation and recycling beyond Earth. They provide evidence that microbial cellular metabolism remains functional in other gravity conditions distinct from Earth, at least for the organisms and process studied. This experiment also showed how a miniature space biomining reactor was successfully used to demonstrate the principle of such a facility on other planetary bodies. This reactor could be upscaled to an industrial size to provide elements such as vanadium for the construction of high quality and strength metal alloys in space to support the extraterrestrial construction industry, as well as the production of nuclear reactor components, batteries, catalysts, superconductors and other high technology products.

## Data Availability Statement

The original contributions presented in the study are included in the article/Supplementary Material, further inquiries can be directed to the corresponding author/s.

## Author Contributions

CC conceived the BioRock experiment in the framework of the ESA topical team Geomicrobiology for Space Settlement and Exploration (GESSE). CC, RS, and KF designed the experiments for this manuscript. NN and C-ML carried out ground experiments and studies in preparation for flight. CC, RS, and AW integrated the hardware for spaceflight and ground controls. CC, RS, and LE produced the experimental data. CC, RS, and JO performed the data analyses. RM, PR, FF, RV, NL, and IC provided *B. subtilis* and *C. metallidurans* samples, respectively. LP performed the procedures onboard the ISS. RD, JH, JK, AK, NC, and LZ supervised the technical organization and implementation of the experiment at ESA. JD-W, MH, and BR supervised the flight procedures. AM, SP, FC, GL, MB, and VZ designed and fabricated the hardware. RE and JW hosted the ground control experiment. CC wrote the manuscript. All authors discussed the results and commented on the manuscript.

## Conflict of Interest

VZ, MB, AM, SP, FC, and GL were employed by the company Kayser Italia S.r.l. The remaining authors declare that the research was conducted in the absence of any commercial or financial relationships that could be construed as a potential conflict of interest.
