# Supplementary material for: Microbially-Enhanced Vanadium Mining and Bioremediation Under Micro- and Mars Gravity on the International Space Station
Source: Front Microbiol. 2021 Apr 1;12:641387. doi: 10.3389/fmicb.2021.641387 (PMC8047202; doi:10.3389/fmicb.2021.641387)
Supplement: Supplementary file 1 [file Table_1.pdf]

frontiers

Microbially-enhanced vanadium mining and bioremediation under micro- and Mars gravity on the International Space Station

Cockell, Santomartino et al., 2021

Supplementary Material

Supplementary Table 1. Bioleaching and control leaching of vanadium from basalt on the International Space Station (ISS) and on Earth. Total quantity (ng in bulk fluid) of vanadium in each of the experimental flight and ground control samples at the end of the experiment for each of the three organisms examined and sterile samples. The numbers of the experimental units are also shown. 'Cont.' means contamination and sample was discarded.

		Sample 1	Unit No.	Sample 2	Unit No.	Sample 3	Unit No.	Mean	sd
S. desiccabilis	**Microgravity**	22.211	133/1	19.668	130/1	17.631	130/2	19.837	2.295
	**Mars gravity**	20.815	143/1	26.213	142/1	23.325	142/2	23.451	2.701
	**Earth gravity**	23.291	137/1	20.931	118/1	20.271	118/2	21.497	1.588
B. subtilis	**Microgravity**	34.062	131/1	30.780	134/1	26.300	134/2	30.381	3.897
	**Mars gravity**	18.890	145/1	28.444	144/1	24.145	144/2	23.826	4.785
	**Earth gravity**	24.907	121/1	19.595	138/1	23.974	138/1	22.825	2.836
C. metallidurans	**Microgravity**	12.371	117/1	12.249	132/1	14.953	132/2	13.191	1.527
	**Mars gravity**	10.278	148/1	12.204	147/1	11.193	147/2	11.225	0.964
	**Earth gravity**	16.511	141/1	9.0560	140/1	14.053	140/2	13.207	3.799
**Non-biological control**	**Microgravity**	13.690	133/2	7.7642	117/2	Cont.	131/2	10.727	4.190
	**Mars gravity**	14.394	143/2	9.9212	145/2	8.2076	148/2	10.841	3.194
	**Earth gravity**	13.344	137/2	9.0497	121/2	8.5081	13412	10.301	2.649
**Ground 1 x *g* experiment**	***S. desiccabilis***	26.909	154/1	21.283	153/1	19.889	153/2	22.694	3.717
	B. subtilis	21.589	156/1	25.477	155/1	21.363	155/2	22.810	2.313
	C. metallidurans	9.5152	158/1	12.367	157/1	8.9014	157/2	10.261	1.849
	**Control (non-biology)**	17.632	154/2	5.7486	158/2	Cont.	156/2	11.690	8.402
